# Expectation Gates Neural Facilitation of Emotional Words in Early Visual Areas

**DOI:** 10.3389/fnhum.2019.00281

**Published:** 2019-08-23

**Authors:** Sophie M. Trauer, Matthias M. Müller, Sonja A. Kotz

**Affiliations:** ^1^Lehrstuhl für Allgemeine Psychologie, Institut für Psychologie, Universität Leipzig, Leipzig, Germany; ^2^Department of Neuropsychology and Psychopharmacology, Faculty of Psychology and Neuroscience, Maastricht University, Maastricht, Netherlands; ^3^Department of Neuropsychology, Max Planck Institute for Human Cognitive and Brain Sciences, Leipzig, Germany

**Keywords:** emotion, reading, visual attention, anticipation, expectation, SSVEP, SPN

## Abstract

The current study examined whether emotional expectations gate attention to emotional words in early visual cortex. Color cues informed about word valence and onset latency. We observed a stimulus-preceding negativity prior to the onset of cued words that was larger for negative than for neutral words. This indicates that in anticipation of emotional words more attention was allocated to them than to neutral words before target onset. During stimulus presentation the steady-state visual evoked potential (SSVEP), elicited by flickering words, was attenuated for cued compared to uncued words, indicating sharpened sensory activity, i.e., expectation suppression. Most importantly, the SSVEP was more enhanced for negative than neutral words when these were cued. Uncued conditions did not differ in SSVEP amplitudes, paralleling previous studies reporting lexico-semantic but not early visual effects of emotional words. We suggest that cueing mediates re-entrant engagement of visual resources by providing an early “affective gist” of an upcoming word. Consequently, visual single-word studies may have underestimated attentional effects of emotional words and their anticipation during reading.

## Introduction

Emotional signals influence perception and behavior as well as their underlying neural activity (Vuilleumier, 2005; Lang and Bradley, 2009). It is less clear where along the visual stream emotional salience amplifies processing and whether this differs for symbolic (e.g., words) or naturalistic (e.g., faces) stimuli. While emotional scenes and faces have robustly captured visual attention in fMRI (Lang et al., 1998; Bradley et al., 2003; Vuilleumier and Huang, 2009; Hindi Attar et al., 2010b) and ERP studies (Doallo et al., 2006; Pourtois and Vuilleumier, 2006), there is rather mixed evidence regarding emotional words (Keil, 2006; Frühholz et al., 2011). It has been argued that words are just less arousing than pictures (Hinojosa et al., 2009) or that visual complexity accounts for different activation patterns in visual cortex (Schlochtermeier et al., 2013).

Complementing behavioral data, event-related potentials (ERPs), and fMRI, steady-state visual evoked potentials (SSVEPs) elicited by flickering stimuli represent a temporally continuous signal that is mainly generated in early visual areas (V1, V3, and V5; Di Russo et al., 2007; Andersen and Müller, 2010). Its amplitude is robustly modulated not only by spatial attention (Morgan et al., 1996; Müller and Hillyard, 2000) but also by the non-spatial allocation of attention to different stimulus features or sensory modalities (Müller et al., 2006; Andersen et al., 2009; Saupe et al., 2009).

Previous studies have shown that emotional rather than neutral scenes and faces enhance the SSVEP amplitude as an index of early visual processing (Keil et al., 2001, 2003; Bakardjian et al., 2011) or draw processing resources from a concurrent flickering foreground task when presented as distractors (Müller et al., 2008; Hindi Attar et al., 2012; Bekhtereva et al., 2015). The latency of these SSVEP emotion effects (∼300 ms) suggests that not the initial feedforward-sweep in visual cortex is enhanced by emotional saliency but that re-entrant feedback amplifies visual (re-)analysis. Supporting this view, Bekhtereva et al. (2015) showed that SSVEP emotion effects follow affective cue extraction as indexed in ERP emotion effects. Comparing faces and scenes they found an earlier ERP modulation and SSVEP distraction effect for faces. Given that faces are more iconic than complex scenes, i.e., their visual features are more closely associated with emotional content, we consider the following suggestion by Barrett and Bar (2009): affective expectations arise simultaneously with the “gist” of objects, scenes, or faces, and aid perception rather than being a consequence of conscious object recognition. This mechanism may explain why early emotion effects are not robustly found for words: words are complex symbolic stimuli and the perceptual “gist” of a word form in low visual frequencies gives little information about word meaning. Accordingly, studies that examined emotional words in SSVEP designs provide rather mixed results: a sustained decrease of the SSVEP amplitude (Koban et al., 2010), a short-lived amplification during the attentional blink (Keil et al., 2006), or no modulation at all (Trauer et al., 2012, 2015).

The aim of the current study was to examine whether context and expectations are a prerequisite for the *visual* amplification of emotional word processing. We therefore used color cues that predicted the onset latency and valence of an upcoming word in a 15 Hz flicker stimulation protocol and compared SSVEP amplitudes elicited by cued and uncued emotional and neutral words. Given the mixed previous results on emotional words, we analyzed SSVEP amplitude time courses rather than fixed time windows. As an indicator of preparatory cue processing, we examined the stimulus-preceding negativity (SPN), a central negative slope in the ERP prior to cued events that varies as a function of expected emotional relevance (Simons et al., 1979; Fuentemilla et al., 2013). In contrast to our previous work (Trauer et al., 2012, 2015), we chose a valence judgment task to probe whether the task-relevance of emotional word meaning is a prerequisite for emotion effects in early visual cortex.

## Materials and Methods

Nineteen volunteers (10 female, mean age 22.5 years, *SD* 2.6) participated and received course credit or monetary compensation (6 €/h). All had normal or corrected-to-normal vision were right-handed, native speakers of German, and self-reported no reading or spelling deficits. The experiment conformed to the Code of Ethics of the World Medical Association and the standards of the local ethics committee of the University of Leipzig.

### Stimuli

**FIGURE 1:**
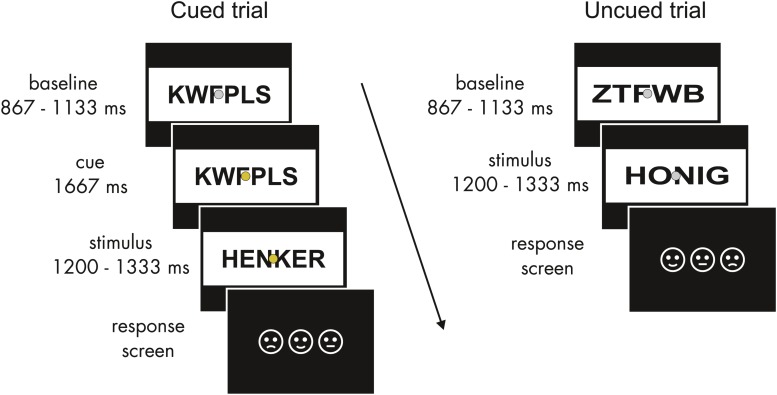
Schematic structure of experimental trials. In cued trials (left), after the baseline period a fixed time period of 1667 ms with a color change of the fixation dot allowed for expectations about the emotional valence of the upcoming word. Uncued trials (right) were identical except for the cue. Baseline and word stimuli flickered at 15 Hz. The response screen indicated the trial-by-trial key assignment.

Word lists comprised four- to six-letter words from the Leipzig Affective Norms for German (LANG; Kanske and Kotz, 2010). 108 words were selected for each emotional category. Positive and negative words had similar arousal ratings (*t*_107_ = 1.2, *p* > 0.2) but differed significantly from neutral words (both *t*_107_ > 20, *p* < 0.001). Valence ratings for all word categories differed significantly from each other (all |*t*_107_| > 20, *p* < 0.001); words were matched for letter and syllable length, print frequency (see Wortschatz Lexikon of the University of Leipzig^1^), and concreteness (all |*t*_107_| < 1.5, all *p* > 0.2). All words were paired with consonant strings that served as flickering baseline stimuli to establish the SSVEP response prior to a word’s onset. Words were presented in black font within a white rectangle of a constant size (15 × 6.5 degrees of visual angle). A word spanned approximately 11 by 4°. In order to control for physical factors influencing SSVEP amplitudes, the luminance of baseline and task stimuli was held constant, and all letter strings were stretched horizontally to comprise a constant number of pixels (Figure 1). A non-flickering fixation dot (0.3°) was superimposed throughout stimulus presentation. Stimuli were presented on a 19-inch CRT monitor at a viewing distance of 80 cm. Word stimuli flickered at 15 Hz realized at a monitor refresh rate of 60 Hz with two frames on and two frames off screen.

### Task and Procedure

Participants gave informed consent and were then seated in a sound-attenuated chamber. Here electrodes were applied and the task was explained. Possible color changes of the fixation dot were pointed out as task irrelevant. Participants were asked to avoid eye movements during the flickering presentation. They were instructed to attentively read the words following a consonant string and to rate them as pleasant, unpleasant, or neutral via a button press. As preparatory activity related to response selection can affect anticipation, three response keys were randomized across trials. Pictogram faces, presented in varying order after the target word presentation, indicated the key assignment for each trial (Figure 1). Key presses were registered starting 200 ms after the onset of the response screen until the response occurred. Participants completed a short training block of 36 additional filler words (also taken from the LANG word lists) to familiarize themselves with the task. The experiment was recorded in 12 blocks of 5 min to allow for breaks.

A consonant string baseline was presented from 867 to 1133 ms. In the cued trials, the color of the fixation dot then changed from gray to one of three colors that were randomly assigned to the three emotional categories and counterbalanced across participants. To avoid differences in salience and prior semantic associations between emotional categories and colors (e.g., red equals negative), isoluminant quasi-complementary secondary colors (best described as mauve, turquoise, and ochre) were used. The cue was presented for a fixed period of 1667 ms. Subsequently, the consonant string was replaced by a word from 1200 to 1333 ms. Except for the cue period, the presentation sequence was the same for uncued trials. Emotional categories and cue conditions were balanced across blocks and randomized within blocks. No more than three consecutive trials of words of the same emotional category were allowed.

Each word was presented three times: in an uncued, validly cued, and a filler condition (with a valid or invalid cue). Due to adaptations of the stimulation program during the first eight recordings, the overall validity ratio of the color cue alternated between 75 and 87.5%, i.e., the ratio (50–75%) of invalidly cued trials differed in the filler condition. We accepted this variation in expectation strength, and focused on the dichotomous effect of validly cued compared to uncued trials; we also discarded the filler trials due to a rather small and inconsistent number of invalidly cued trials. Accordingly, we included data of the first eight participants in the full data analysis. The subsequent 11 recordings were conducted with a set cue validity of 83.3%. The order of uncued, validly cued, or filler trials was counterbalanced across words. We analyzed the uncued and the first validly cued presentation of each word. There were 108 experimental trials in each of the 6 experimental conditions [*uncued*/(*validly*) *cued* by *neutral*/*negative*/*positive word*].

### EEG Data

**FIGURE 2:**
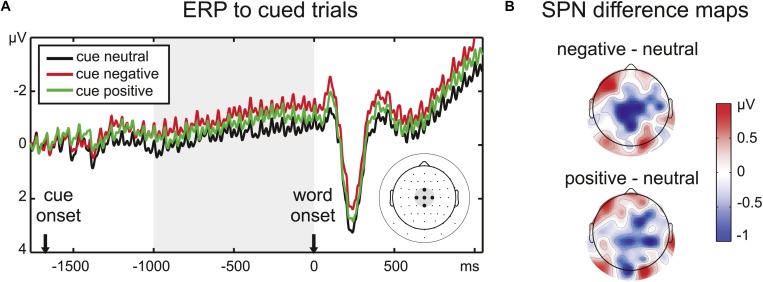
**(A)** ERP time course at the five central electrodes chosen to analyze the SPN amplitude, indicated on the scalp electrode map at the right side of the panel. Note that condition effects continue after the cue period throughout the stimulus period. **(B)** Difference maps for the SPN time window (1000 ms before word onset) reveal a larger central negativity prior to emotional words, especially negative words (upper scalp map).

**FIGURE 3:**
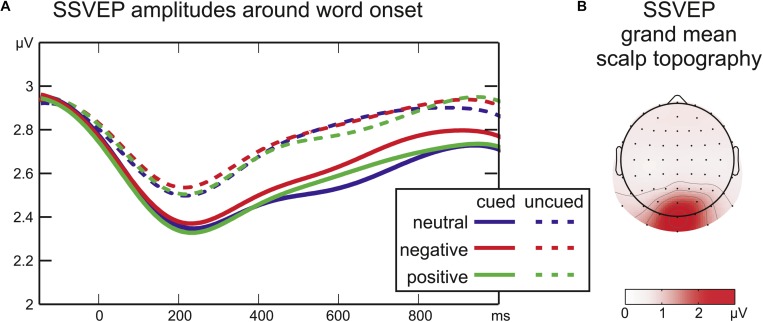
**(A)** Time courses of SSVEP amplitude after word onset. The cue period was analyzed separately (cued trials only, not depicted). **(B)** Grand mean SSVEP amplitude across all conditions from –350 to 1200 ms around word onset.

The electroencephalogram (EEG) was recorded from 64 Ag/AgCl scalp electrodes (see Figures 2, 3 for the electrode layout) referenced to “common mode” at a sampling rate of 256 Hz using an ActiveTwo amplifier system (BioSemi, Amsterdam). Four additional electrodes recorded the horizontal and vertical electrooculogram. EEG data were processed using the ERPLAB plugin^2^ (Delorme and Makeig, 2004) running on MATLAB. Epochs were extracted from −500 to 1200 ms around the word onset for uncued trials and from −2167 to 1200 ms for cued trials. Due to the expected ERP variation during cue presentation, cued trials were baseline corrected to the time range of 100 ms before cue onset. Uncued trials were baseline corrected to 100 ms before word onset. Thus, the stimulation during the baseline period was identical. Trials with eye movements or blinks were excluded. Artifacts such as noisy electrodes were corrected using a combination of channel approximation and epoch exclusion based on statistical parameters of the data with the “statistical control of artifacts in dense array EEG/MEG studies” (SCADS; Junghöfer et al., 2000). Corrected data were then re-referenced to the average signal across all electrodes. Data of two participants were discarded because more than one-third of EEG data epochs contained eye movements or muscle artifacts. For the remaining 17 datasets on average 6.25% of trials were excluded, and 3.28 channels per trial were interpolated. For each condition and individual the amplitudes of all trials were averaged for further analyses.

### Stimulus-Preceding Negativity (SPN) Analysis in Cued Trials

The ERPs were analyzed in the same dataset as the SSVEP amplitudes. A time range of 1000 ms before word onset was defined as the SPN time window by visual inspection of the grand mean amplitude. The slow negativity during this time range peaked at centroparietal electrodes, therefore, a cluster of five central electrodes was chosen for the analysis of the SPN (Figure 2). Amplitudes averaged across the chosen time window and electrodes were analyzed with a repeated-measures ANOVA with the factor *Emotion*.

We refrained from analyzing later ERP components as we did in previous studies (Trauer et al., 2012, 2015) as the cue period altered the baseline for cued and uncued trials, and the SPN deflection persisted throughout the trial (Figure 2) and superimposed the ERP to the word onset in cued trials.

### Steady-State Visual Evoked Potential (SSVEP) Analyses

The average amplitude of the 15 Hz signal from −350 to 1050 ms around word onset was calculated at each electrode using a Fourier transformation (implemented as “fft” in Matlab). For each participant the three occipital electrodes exhibiting highest SSVEP amplitudes across all conditions were selected. For each participant and condition the averaged signal at these three individual electrodes served to extract the time courses of the SSVEP amplitude by means of a Gabor Filter centered at 15 Hz with a frequency resolution of ±1.47 Hz full-width at half-maximum and a temporal resolution of ±150 ms. For cued conditions the pre-stimulus period was analyzed separately from −1667 to 0 ms. Given that SSVEP amplitude is unaffected by baseline shifts in the ERP, the SSVEP amplitudes after word onset (0–1050 ms) of all experimental conditions were entered into a two-factorial ANOVA (*Cue: uncued/cued* by *Emotion: neutral/negative/positive*) at each sampling point. Regarding the resulting large number of tests, instead of a conservative correction for multiple comparisons, effects were only regarded relevant when statistical significance was reached for 10 or more subsequent data points [39 ms, see Andersen and Müller (2010) for a similar approach]. All significant effects in the analyses met this criterion, i.e., all detected differences are reported below.

## Results

### Behavioral Data

Rating responses for each condition were transformed to percent of ratings agreeing with the affective valence category (Table 1). Across all conditions, participants rated 75.51% of the words according to their assigned affective category. The cue had no influence on rating agreement (*F*_1__,__16_ = 0.3, *p* > 0.5). There was no significant influence of affective valence on rating agreement (*F*_2__,__32_ = 0.6, *p* > 0.5), although rating agreement was slightly higher for negative words.

**TABLE 1:** Rating responses agreeing with the preselected emotional category in percent.

	**No cue**			**Valid cue**		
		
**Word set**	**Neutral**	**Negative**	**Positive**	**Neutral**	**Negative**	**Positive**
**% Responses**						
Neutral	**73.12**	15.55	22.35	**72.12**	15.93	21.16
Unpleasant	6.29	**79.37**	3.16	6.12	**78.45**	3.34
Pleasant	20.59	5.08	**74.49**	21.76	5.62	**75.50**
		
Sum % matches	75.66			75.35		
	75.51					

*Each column represents one experimental condition and sums up to 100%. Bold numbers indicate responses according to the preselected emotional category.*

**FIGURE 4:**
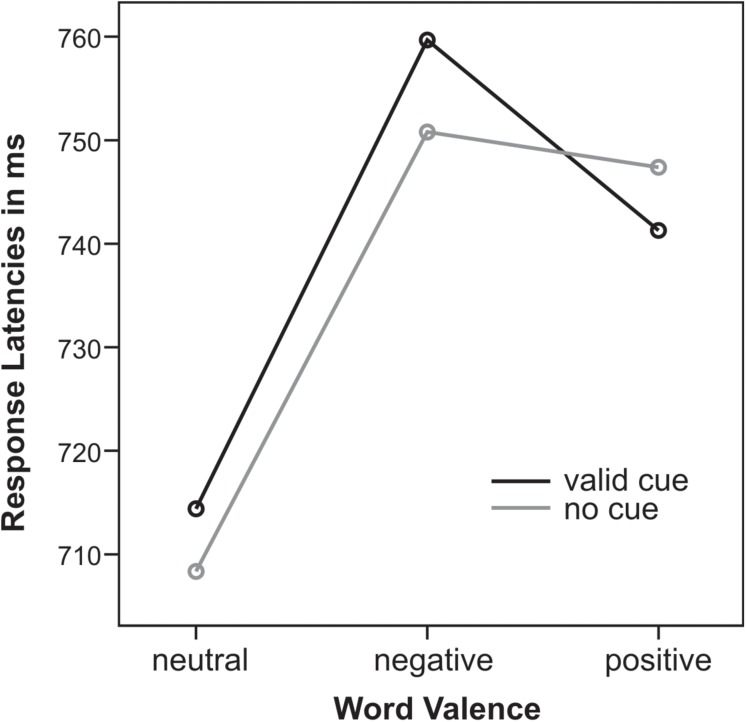
Response latencies in ms for valid cues (black) and no cues (gray) for neutral, negative, and positive words.

Response times (RTs, mean 736 ± 77 ms, locked to the onset of the response screen) were delayed from stimulus onset by a trial-by-trial key assignment. There was a main effect of emotional word content (*F*_2__,__32_ = 14.3, *p* < 0.001, ${\text{η}}_{\text{p}}^{2}$ = 0.47): for both cueing conditions emotional positive and negative words led to slower responses than neutral words (all *t*_16_ > 2.8, all *p* < 0.01). There was a marginal trend for an interaction of *Cue* and *Emotion* (*F*_2__,__32_ = 2.7, *p* = 0.1): only for uncued trials RTs for negative compared to positive words were significantly slower (*t*_16_ = 3.4, *p* < 0.01) whereas for cued trials RTs did not differ (*t*_16_ = 0.6, *p* = 0.6). This *Cue* by *Emotion* interaction was closer to significance when only trials with valence rating agreement were analyzed (*F*_2__,__32_ = 3.2, *p* = 0.06, ${\text{η}}_{\text{p}}^{2}$ = 0.17). Note that next to this trend interaction, cueing had no effect on response latencies (Figure 4).

### Stimulus-Preceding Negativity (SPN)

There was a significant effect of the implicit valence of the color cue (*F*_2__,__32_ = 5.17, *p* < 0.05, ${\text{η}}_{\text{p}}^{2}$ = 0.24) on the pre-stimulus ERP amplitude at the central electrode cluster. Pairwise comparisons confirmed an enhanced SPN for negative compared to neutral cues (*t*_16_ = 3.05, *p* < 0.01). There was also a trend toward a larger SPN for positive compared to neutral cues (*t*_16_ = 1.83, *p* = 0.09). SPN amplitudes for positive and negative cues did not differ significantly (*t*_16_ = 1.50, *p* > 0.1).

### Steady-State Visual Evoked Potential (SSVEP)

During the cue period, there was no significant effect of *Emotion* on SSVEP amplitudes (all *F*_2__,__16_ < 1.1; all *p* > 0.2; not depicted). Moreover, in all cued conditions the SSVEP amplitude remained stable at all sampling points compared to a baseline of 100 ms before cue onset (all |*t*_16_| < 1.3; all *p* > 0.1).

After word onset the factor *Cue* was significant for the SSVEP amplitude from 215 ms until the end of the analyzed time range at 1050 ms (all *F*_1__,__16_ > 4; all *p* < 0.05) reflecting a larger drop in SSVEP amplitude after word onset for cued compared to uncued words (Figure 3). Later in the stimulus period, a significant interaction of *Cue* by *Emotion* (508–629 ms, all *F*_2__,__32_ = 3.2, all *p* < 0.05) was followed by a significant main effect of *Emotion* (648–723 ms, all *F*_2__,__32_ > 3.3, all *p* < 0.05). SSVEP time-courses of cued and uncued conditions were analyzed separately with one-factorial repeated-measures ANOVAs with the factor *Emotion* to disentangle interaction effects. For uncued words no effect of emotional word content was found (at all sampling points *F*_2__,__32_ < 1.9, all *p* > 0.18). For cued words there was a significant main effect of *Emotion* from 555 to 742 ms (all *F*_2__,__32_ > 3.4; all *p* < 0.05). Running *t*-tests confirmed higher SSVEP amplitudes for cued negative compared to cued neutral words from 320 to 828 ms (all *t*_16_ > 4.4, all *p* < 0.05). There was a trend toward higher amplitudes for cued negative compared to cued positive words around 730 ms [max(*t*_16_) = 3.7, min(*p*) = 0.07]. SSVEP amplitudes elicited by cued positive and cued neutral words did not differ significantly (all *t*_16_ < 1.8, all *p* > 0.2).

## Discussion

Is anticipation a prerequisite for emotional words to capture attention early in the visual stream? The present work set out to answer this question. In line with our hypothesis that an emotional “gist” provided by a valence cue can gate an emotion effect in occipital cortex, we report a more enhanced SSVEP amplitude in response to negative than neutral words in the cued but not in the uncued conditions.

### Affective Expectation Reflected in SPN Amplitude

Prior to word onset, a subtle color cue was sufficient to set off anticipation of emotional word content indexed by the SPN amplitude modulation. One limitation in the interpretation of this result is that we did not assess explicit learning of the color cue and also could not clearly track the process of cue learning, e.g., by comparing the first and the second half of the experiment due to our presentation randomization. Future studies may use a more suitable design to track such learning curves and the associated anticipation effects with emotional words on preparatory activity and subsequent visual attention and performance effects. However, the significant SPN effect reported here indicates that throughout the experiment, implicitly or explicitly, the cue colors acquired differential affective meaning for the participants. Anticipatory effects of emotional stimuli on SPN deflections have been reported in several tasks (Simons et al., 1979; Regan and Howard, 1995; Böcker et al., 2001; Poli et al., 2007; Fuentemilla et al., 2013; Michalowski et al., 2015), even though findings are mixed regarding the direction of emotion effects on the SPN amplitude (decreased prior to aversive stimuli: Lumsden et al., 1986; Judah et al., 2013). Yet, to our knowledge, this is the first demonstration of an SPN emotion effect for written words. Emotional valence was the task-relevant dimension, but the assignment of the response keys was presented only after the word offset. Therefore, the SPN slope observed in the present study is unlikely to reflect mere motor preparation (Birbaumer et al., 1990; Brunia et al., 2011). This task design could also be the reason why the affective SPN modulation at central electrodes persisted throughout word presentation: final response selection was only possible after a word’s offset. Continuity of the SPN may thus represent the maintenance of cue and word information, as suggested by Dias et al. (2003) who linked the SPN offset latency to reaction times rather than the target onset. In line with prior research (Judah et al., 2013; Schevernels et al., 2014) this negative shift seems to reflect anticipatory mobilization of neural resources for impending cognitive effort. This notion is supported by generally longer response latencies for emotional than neutral words. However, the cue and enhanced SPN activity prior to emotional words did not lead to an RT benefit for cued compared to uncued negative words, indicating that cueing may have even enhanced rather than dissolved disengagement from negative stimuli or conflict between stimulus processing and task processing.

Although the SPN amplitudes to positive and neutral words did not differ significantly, both positive and negative words led to a numerically larger pre-stimulus negativity, suggesting that arousal likely enhanced anticipatory processing. The more distinct ERP modulation for negative words may be related to the slightly higher rating agreement for the negative category (Table 1), indicating that negative words are perceived as less ambiguous.

### Expectation Suppression Reflected in SSVEP Amplitudes

Steady-state visual evoked potential amplitudes did not vary during the cue presentation, indicating that the visual processing of letter strings was not modulated before stimulus onset. This parallels previous imaging results, revealing no additional activation of posterior occipital areas during the anticipation of emotional pictures, but dissociable networks for anticipation and perception of emotion (Simmons et al., 2004; Bermpohl et al., 2006).

At word onset SSVEP amplitudes elicited by cued compared to uncued words showed a larger drop and remained lower for the full duration of the stimulation period. This finding may seem surprising, given that the cue allowed for the precise temporal allocation of attention, and attention has been shown to enhance SSVEP amplitudes (Morgan et al., 1996; Müller and Hillyard, 2000). However, these studies used spatial rather than temporal cues. Temporal and spatial orienting seem to exert separable and interactive effects: temporal expectations alone lead to behavioral facilitation, but early visual activity, as reflected in the P1 component, is modulated by temporal cueing only when stimulus location is also predictable (Doherty et al., 2005; Rohenkohl et al., 2014). Stimulus location was fixed in the present study, but SSVEP amplitude revealed no additional amplification as was reported for the P1. Therefore, the present results are more in line with the framework of predictive coding (Rao and Ballard, 1999): Anticipation presumably silences the signals of predictable sensory input via re-entrant feedback. Accordingly, activity in visual cortex has been found reduced for predictable stimuli (unattended: den Ouden et al., 2009; passive viewing: Alink et al., 2010; attended: Kok et al., 2012b). Predictions may “sharpen” patterns of activity in non-spatial tasks leading to effective and less ambiguous sensory signals (Kok et al., 2012a). Such a mechanism may be reflected in attenuated SSVEP amplitudes for the cued words in the present study.

### SSVEP Emotion Effect for Cued Words Only

Attention, in contrast to prediction, is defined as the sensory amplification of salient or relevant events. How anticipation and attention overlap and interact, however, remains a matter of debate (Summerfield and de Lange, 2014; Schröger et al., 2015). In visual V1 and V3 cortices, the main generators of the SSVEP (Di Russo et al., 2007; Andersen and Müller, 2010), voluntary attention can reverse the attenuation of predicted signals (Kok et al., 2012b). Could emotional relevance result in similar attentional effects? On the one hand, all words in the current study were task-relevant and thus attended. In contrast to the study by Kok et al. (2012b), predictable stimuli still elicited smaller SSVEP amplitudes, perhaps because the task required a semantic rather than a visual choice. However, an additional attentional bias toward aversive stimuli may have reversed the attenuation of visual activity resulting in a relative enhancement of SSVEP amplitude for cued negative words. This was not found for uncued words. In line with the “gist” hypothesis of Barrett and Bar (2009), we argue that the color cue signaled emotional valence and may have gated a re-entrant attention effect of emotional words in early visual cortex. Such a re-entrant effect seems to be weaker or absent when emotional words are presented without a cue (Trauer et al., 2012, 2015).

Paralleling previous studies using flickering IAPS pictures (Keil et al., 2003; Hindi Attar et al., 2012) or expressive faces (Bakardjian et al., 2011; McTeague et al., 2011; Bekhtereva et al., 2015), the SSVEP amplitude increase indicates that negative words received more visual processing resources. Examining affective stimuli, time-courses of the SSVEP amplitude have mostly been reported in distraction studies, i.e., when the SSVEP elicited by a foreground task is assessed while emotional distractors are presented in the background. Despite of this methodological difference, the latency of the emotion effect of cued negative words here (320–828 ms) roughly matches the time course of distraction by emotional background scenes (Schönwald and Müller, 2014: ∼350–700 ms; Hindi Attar et al., 2010a: ∼400–1000 ms; Müller et al., 2008: ∼400–1000 ms) indicating a similar underlying attention effect.

The latency of the SSVEP effect also renders it likely that word content rather than cue valence alone led to the attentional enhancement in visual processing. Additionally, the SSVEP effect was driven by negatively valenced words alone, and thus did not fully parallel the valence effect on the SPN amplitude. However, only validly cued and uncued trials were analyzed due to the small number of invalidly cued trials. Further experiments should disentangle the contributions of cue valence and target valence by orthogonally manipulating the two factors. Additionally, correlating cue-related activity (e.g., the SPN) and visual activity related to the target word (here the SSVEP) in further studies could shed more light on the underlying mechanisms of expectation and subsequent emotional facilitation.

In contrast to previous studies the emotional valence of words was task-relevant. However, given that no effect was found for uncued but equally task-relevant emotional words, expectancy rather than task-relevance seemed to be crucial for the observed SSVEP modulation.

The current result is novel compared to previous reports of SSVEP amplitude modulation by emotional words. Keil et al. (2006) reported an SSVEP amplitude increase for negative words specifically when they were presented during the attentional blink. Did their design allow for an anticipation account? Neutral first targets (T1) in the rapid serial visual stream predicted that second targets (T2) would occur with an onset asynchrony of 232–696 ms and T2 were arousing words with a probability of 66%. These regularities are rather loose and would also not account for the absence of an emotion effect for T2 presented more than 232 ms after the T1. Additionally, the latency of the SSVEP effect (120–270 ms) does not correspond to the emotion effect found here or in SSVEP studies using emotional scenes. We assume that the rapid serial presentation may reflect different attentional demands compared to the present experiment. Koban et al. (2010) did not report SSVEP amplitudes during the first second of stimulation, thus the late amplitude decrease for positive words during free viewing in their study cannot be related to the earlier effect found here. Previous experiments from our laboratory (Trauer et al., 2012, 2015) found no influence of emotional word content on SSVEP amplitudes during lexical decision or distraction from a visual foreground task. In both studies the latency of word onset as well as emotional word valence were not predictable. In line with these prior findings no SSVEP effect of uncued negative words was evident in the present experiment, strengthening the notion that written emotional words do not capture additional visual resources by default. However, when a cue or context allows for emotional expectations, written emotional words may exert similar effects on early visual processing as pictorial stimuli.

## Data Availability

The raw data supporting the conclusions of this manuscript will be made available by the authors upon reasonable request by any qualified researcher.

## Ethics Statement

This study was carried out in accordance with the recommendations of the “University of Leipzig, Ethics committee” with written informed consent from all subjects. All subjects gave written informed consent in accordance with the Declaration of Helsinki. The protocol was approved by the “Ethics committee of the University of Leipzig, Germany.”

## Author Contributions

All authors were involved with the design and interpretation of the study. ST programmed and conducted the experiment, analyzed the data, and drafted the manuscript and figures. MM revised the manuscript. SK revised and edited the final manuscript.

## Conflict of Interest Statement

The authors declare that the research was conducted in the absence of any commercial or financial relationships that could be construed as a potential conflict of interest.

## Footnotes

1 http://wortschatz.uni-leipzig.de

2 http://erpinfo.org/erplab
